# *Limosilactobacillus fermentum* KBL674 Alleviates Vaginal Candidiasis

**DOI:** 10.1007/s12602-024-10403-3

**Published:** 2024-11-19

**Authors:** Sung Jae Jang, Eun Jung Jo, Cheonghoon Lee, Bo-Ram Cho, Yun Jeong Shin, Jun Soo Song, Woon-Ki Kim, Nanhee Lee, Hyungjin Lee, SungJun Park, GwangPyo Ko

**Affiliations:** 1 Department of Environmental Health Sciences, Graduate School of Public Health, Seoul National University, Seoul, Republic of Korea; 2 weBiom Inc., Seoul, Republic of Korea; 3 KoBioLabs, Inc, Seoul, Republic of Korea; 4 Institute of Health and Environment, Seoul National University, Seoul, Republic of Korea; 5 The Food Industry Promotional Agency of Korea, Iksan-Si, Jeollabuk-Do Republic of Korea; 6 N-Bio, Seoul National University, Seoul, Republic of Korea

**Keywords:** Candidiasis, Gut microbiota, *Limosilactobacillus fermentum*, Probiotics, Vaginal health

## Abstract

*Candida* *albicans* (*C. albicans*) is the primary etiologic agent of vaginal candidiasis. *Lactobacillus* species are predominant in the vaginal microbiome; they inhibit the development of vaginal candidiasis by producing antimicrobial agents, such as lactic acid and hydrogen peroxide. In this study, we investigated the effects of *Limosilactobacillus fermentum* (*L. fermentum*) KBL674 in a mouse model of vaginal candidiasis. *L. fermentum* KBL674 inhibited *C. albicans* hyphal growth. Moreover, oral administration of *L. fermentum* KBL674 significantly suppressed vaginal *C. albicans* infection and associated symptoms, including tissue thickness and immune cell infiltration. A substantial quantity of *L. fermentum* KBL674 was excreted by the mice within 6 h after oral administration, indicating that most *L. fermentum* KBL674 did not settle within the gastrointestinal tract. *L. fermentum* KBL674 modulated gut microbiome diversity, increasing abundances of the genera *Akkermansia*, *Eubacterium*, and *Faecalibaculum* and family *Muribaculaceae*. Abundances of these bacteria showed negative correlations with the vaginal *C. albicans* burden in the mouse model, suggesting links between the gut microbiome composition and the vaginal *C. albicans* burden*.* Therefore, *L. fermentum* KBL674 can reduce the vaginal *C. albicans* burden via direct or indirect inhibition and modulation of the gut microbiome composition preventively.

**Graphical Abstract:**

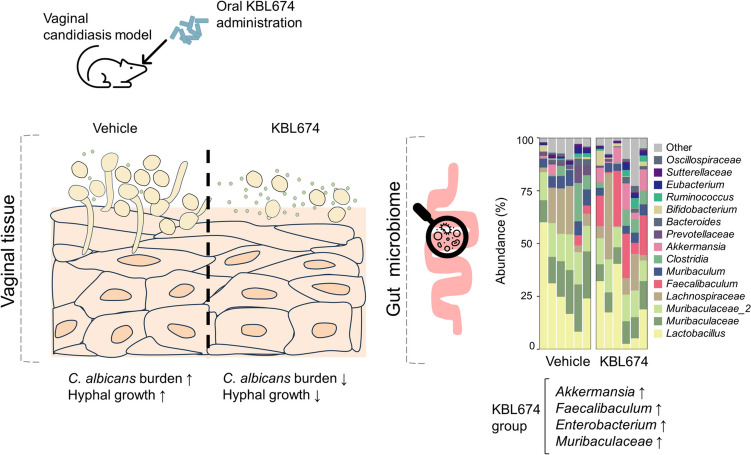

## Introduction

*Candida* species are the most common cause of fungal infection within the vagina. Globally, approximately 75% of women experience vaginal candidiasis at least once in their lifetime; the recurrence rate is 6 to 9% [1]. *Candida albicans* (*C. albicans*) is a dominant yeast species in the vagina [2]. Antifungals (e.g., clotrimazole and fluconazole) are standard treatments for *C. albicans* infection but have various side effects, including allergic responses, toxicities, and increased drug resistance [3, 4]. There are no effective methods of preventing or treating vaginal *C. albicans* infection without side effects.

*Lactobacillus* species are predominant in the vaginal microbiome. A substantial proportion of the genus *Lactobacillus* is present in healthy women of reproductive age. Moreover, *Lactobacillus* species inhibit vulvovaginitis by producing antimicrobial agents (e.g., lactic acid and hydrogen peroxide) [5–7]. Microbial communities with low abundances of *Lactobacillus* species are strongly correlated with genital inflammation, which exerts a negative effect on reproductive health and can increase the risk of human immunodeficiency virus infection [8, 9]. Moreover, abundances of the genera *Gardnerella* and *Ureaplasma*, major vulvovaginitis pathogens, are elevated in the *Lactobacillus*-deficient vagina [10]. These findings emphasize the importance of *Lactobacillus* species in preventing vaginal infection.

Commercially available probiotics containing vagina-derived *Lactobacillus* species, including *Lactobacillus crispatus*, *Lacticaseibacillus rhamnosus*, and *Limosilactobacillus reuteri*, have been suggested for vaginal health maintenance and vaginal candidiasis prevention [11–13]. However, there is a lack of supporting clinical evidence. Our previous study has suggested that the conditioned supernatant from *Limosilactobacillus fermentum* (*L. fermentum*) KBL674, isolated from the vagina of a healthy Korean woman, inhibited the growth and hyphae formation of *C. albicans* [14]. Therefore, *L. fermentum* KBL674 shows promise as a probiotic for preventing vaginal candidiasis.

In this study, we investigated the effects of *L. fermentum* KBL674 on *C. albicans* infection using in vitro and in vivo models. We evaluated fecal excretion of *L. fermentum* KBL674 after oral administration. Additionally, we analyzed the correlations of the vaginal *C. albicans* burden and gut microbiome composition, aiming to investigate the mechanisms by which *L. fermentum* KBL674 inhibits *C. albicans*.

## Materials and Methods

### Preparation of Microorganisms

*C. albicans* (MYA-4788) was purchased from the American Type Culture Collection (ATCC) and cultured using the ATCC method with some modifications [14]. Briefly, *C. albicans* was cultured in Yeast Extract-Peptone-Dextrose medium (10 g/L yeast extract, 20 g/L peptone, and 20 g/L d-glucose) at 30 °C for 18 h at 200 rpm in a shaking incubator and stored at 4 °C until use.

The lyophilized powder of *L. fermentum* KBL674 was provided by weBiom Inc. (Seoul, Republic of Korea). The lyophilized powder without *L. fermentum* KBL674 was used for the vehicle group. Phosphate-buffered saline (PBS; 1 ×) was used to disperse the lyophilized powder. *L. fermentum* KBL674 concentrations measured using the cultivation method are presented as colony-forming units (CFUs).

### Inhibition of hyphal growth of *C. albicans*

Inhibition of *C. albicans* hyphal growth was assessed by analysis of hyphal length [14]. First, cultured *C. albicans* cells were washed twice using 1 × PBS and re-suspended in serum-free Roswell Park Memorial Institute (RPMI) 1640 medium. Subsequently, 100 µL *C. albicans* suspension (5 × 10^4^ cells) were co-cultured with 200 µL *Lactobacillus* culture (20% v/v) in a 24-well culture plate with 700 µL RPMI 1640 medium. The plate was placed in an incubator for 3 h at 37 °C with 5% CO_2_, and the medium was discarded. *C. albicans* cells were fixed with 4% paraformaldehyde and stained with Calcofluor White (Sigma-Aldrich, St. Louis, MO, USA). Hyphae were imaged using a Ti-2E inverted microscope (Thermo Fisher Scientific, Waltham, MA, USA) and hyphal length was measured using the ImageJ software (National Institutes of Health, Bethesda, MD, USA) [15]. All hyphal branches were included in length measurements.

### Mouse Model of Vaginal Candidiasis

Animal experimental procedures were approved by the Institutional Animal Care and Use Committee (IACUC) (Case Number: IACUC-22–017) of The Food Industry Promotional Agency of Korea (Iksan-si, Jeollabuk-do, Republic of Korea). Five-week-old female C57BL/6 mice (16–20 g; Orient Bio, Seongnam-si, Gyeonggi-do, Republic of Korea) were housed under a 12/12 h light/dark cycle under specific pathogen-free conditions. Mice had ad libitum access to sterile food and water; they were acclimated for ≥ 1 week prior to being used in experiments.

The vaginal candidiasis model was established as previously reported with some modifications (Fig. 2A) [16, 17]. First, 100-µL sesame oil containing 0.5 mg β-estradiol 17-valerate (Sigma-Aldrich) was intraperitoneally administered on days 9 and 3 before *C. albicans* infection to maintain a pseudo-estrous condition. Oral administration of *L. fermentum* KBL674 (2 × 10^9^ CFUs) was performed daily from day 7 pre-infection to day 14 post-infection. On the day of infection, 10 µL 1 × PBS containing 5 × 10^6^ *C. albicans* cells was intravaginally administered. On day 15 post-infection, mice were anesthetized and vaginal *C. albicans* burdens were measured using the cultivation method. Mouse ceca, feces, and vaginal tissues were harvested for further experiments.

### Histological Analyses

Histological analysis was performed by T&P Bio (Gwangju-Si, Gyeonggi-do, Republic of Korea). Briefly, vaginal tissues were fixed with 10% neutral-buffered formalin for 24 h and embedded in paraffin. Next, tissues were sectioned and subjected to hematoxylin and eosin (H&E) or periodic acid-Schiff (PAS) staining for histological evaluation of vaginal *C. albicans* infection. The presence and severity of *C. albicans* infection in vaginal tissues were evaluated by analyzing tissue thickness, inflammatory infiltration, and hyperkeratosis.

### Fecal Excretion of *L. fermentum* KBL674

Five-week-old female C57BL/6 mice (16–20 g; Orient Bio) were prepared and acclimated for ≥ 1 week. Next, 100 µL sesame oil containing 0.5 mg β-estradiol 17-valerate (Sigma-Aldrich) was intraperitoneally administered to maintain a pseudo-estrous condition. On day 3 post-injection, the mice were orally administered *L. fermentum* KBL674 (2 × 10^9^ CFUs) (Fig. 3A); fecal samples were collected after 0, 2, 6, and 24 h. The samples were stored at − 80 °C until use.

Bacterial DNA was extracted from fecal samples using a QIAamp DNA Stool Mini Kit (Qiagen, Hilden, Germany), in accordance with the manufacturer’s instructions. The full-length 16S ribosomal RNA (16S rRNA)-coding gene of KBL674 was amplified using primers 27F and 1492R [18] (Table 1) and ligated into a pGEM-T Easy vector (Promega, Madison, WI, USA) to establish a reference standard for quantification. Subsequently, DNA from *L. fermentum* KBL674 was measured by quantitative polymerase chain reaction (qPCR) using a Power SYBR Green PCR Master Mix (Thermo Fisher Scientific) and a QuantStudio 5 Real-Time PCR System (Thermo Fisher Scientific). qPCR was performed with the following thermocycler protocol: denaturation at 95 °C for 10 min, followed by 40 cycles of 95 °C for 15 s and 55 °C for 60 s. Cycle threshold values based on the DNA standard were calculated to determine the copy number of *L. fermentum* KBL674 in 5 ng DNA.

**Table 1:** Sequences of primers used for PCR

Gene	Sequence (5′ → 3′)	Reference
16S rRNA	Forward (27F): 5′-AGRGTTYGATYMTGGCTCAG-3′	[18]
	Reverse (1492R): 5′-RGYTACCTTGTTACGACTT-3′	
*L. fermentum* specific	Forward: 5′-TGAAGAAGGGTTTCGGCTCG-3′	This study
	Reverse: 5′-GCACGTAGTTAGCCGTGACT-3′	
V3-4 region of 16S rRNA	Forward (341F): 5′-CCTACGGGNGGCWGCAG-3′	[20]
	Reverse (805R): 5′-GACTACHVGGGTATCTAATCC-3′	

### Fecal and Cecal Microbiota Analyses

Samples were collected and analyzed as previously described, with some modifications [19]. First, total DNA was extracted from fecal and cecal samples using a FastDNA Stool Mini Kit (Qiagen), in accordance with the manufacturer’s instructions. The V3–V4 region of the 16S rRNA gene [20] was amplified using universal bacterial primers (Table 1). Barcoded amplicons were pooled in equimolar concentrations and sequenced on an Illumina MiSeq platform (Illumina, San Diego, CA, USA) with a MiSeq Reagent Kit v. 3 (Illumina). Raw reads were filtered and trimmed using Trimmomatic v. 0.38 (USEDEL lab; https://github.com/usadellab/Trimmomatic) [21]. Quantitative Insights into Microbial Ecology (QIIME) 2 v. 2024.5 (QIIME 2 Development Team; https://qiime2.org) [22] and the Silva v. 138.1 database (SILVA rRNA database project; https://www.arb-silva.de) [23] were used for taxonomic classification. The Divisive Amplicon Denoising Algorithm 2 was used to demultiplex the reads, followed by error correction and chimera removal. Shannon indices or principal coordinates analysis (PCoA) plots with Bray–Curtis and weighted UniFrac distances were generated to measure the alpha and beta diversities, respectively, using the q2-diversity plugin and qiime2R (QIIME 2 Development Team) [24].

### Statistical Analysis

Data are presented as means ± standard errors (SEM) of at least three independent experiments. When appropriate, data were analyzed by the Mann–Whitney *U* test. *P*-value (*p*) < 0.05 was considered statistically significant. Prism v. 10 (GraphPad Software, San Diego, CA, USA) was used for statistical analysis and visualization. Pearson’s correlation coefficients (*r*) for the vaginal *C. albicans* burden and gut microbiome composition were calculated using R v. 4.1.1 (The R Project; https://www.r-project.org).

## Results

### Effect of *L. fermentum* KBL674 on *C. albicans* Hyphal Growth

**Fig. 1:**
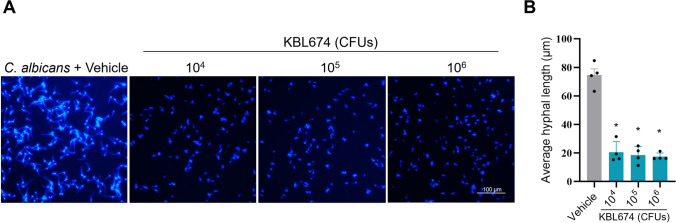
Effect of *L. fermentum* KBL674 on hyphal growth of *C. albicans*. **A** Hyphal growth of *C. albicans*. *C. albicans* was incubated with *L. fermentum* KBL674 in an incubator for 3 h at 37 °C with 5% CO_2_. Subsequently, yeast cells were fixed and stained with calcofluor white. Hyphal growth was visualized by fluorescence microscopy (scale bar: 100 μm). **B** Average hyphal length of *C. albicans*. When appropriate, data are expressed as means ± SEM. Asterisks indicate statistically significance (*p < 0.05; Mann–Whitney *U* test)

*L. fermentum* KBL674 inhibited *C. albicans* hyphal growth in a dose-dependent manner (Fig. 1A). Compared with the vehicle group, the average hyphal length of *C. albicans* was significantly decreased by the lowest concentration of *L. fermentum* KBL674 (1 × 10^4^ CFUs) (*p* < 0.05) (Fig. 1B).

### Effect of *L. fermentum* KBL674 on the Vaginal *C. albicans* Burden

**Fig. 2:**
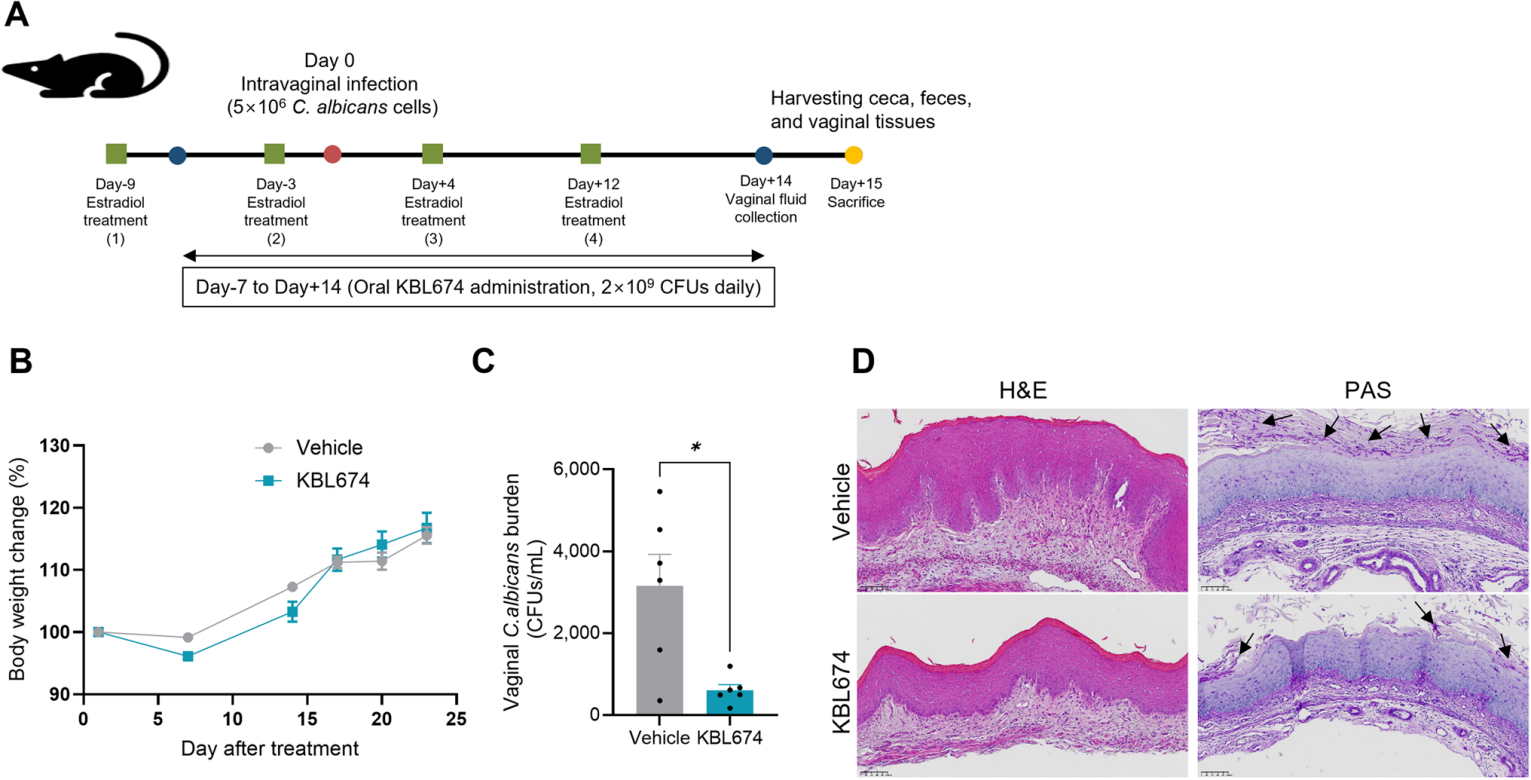
Effect of *L. fermentum* KBL674 in a mouse model of vaginal candidiasis. **A** The experimental scheme of the vaginal candidiasis model. The lyophilized powder without *L. fermentum* KBL674 was used for the vehicle group. **B** Body weight change. **C** Vaginal *C. albicans* burden. **D** Histological evaluation of vaginal *C. albicans* infection (scale bar: 100 μm). Black arrows indicate *C. albicans* in vaginal tissues. When appropriate, data are expressed as means ± SEM. Asterisks indicate statistically significance (*p < 0.05; Mann–Whitney *U* test)

*L. fermentum* KBL674 administration did not affect mouse body weight (Fig. 2B). The vaginal *C. albicans* burden was significantly reduced in the *L. fermentum* KBL674-treated group (Fig. 2C). H&E and PAS staining indicated that the vaginal tissues of the *L. fermentum* KBL674-treated group showed reductions in *C. albicans* infection and associated symptoms, such as tissue thickness and immune cell infiltration, compared with the vehicle group (Fig. 2D).

### Excretion of *L. fermentum* KBL674

**Fig. 3:**
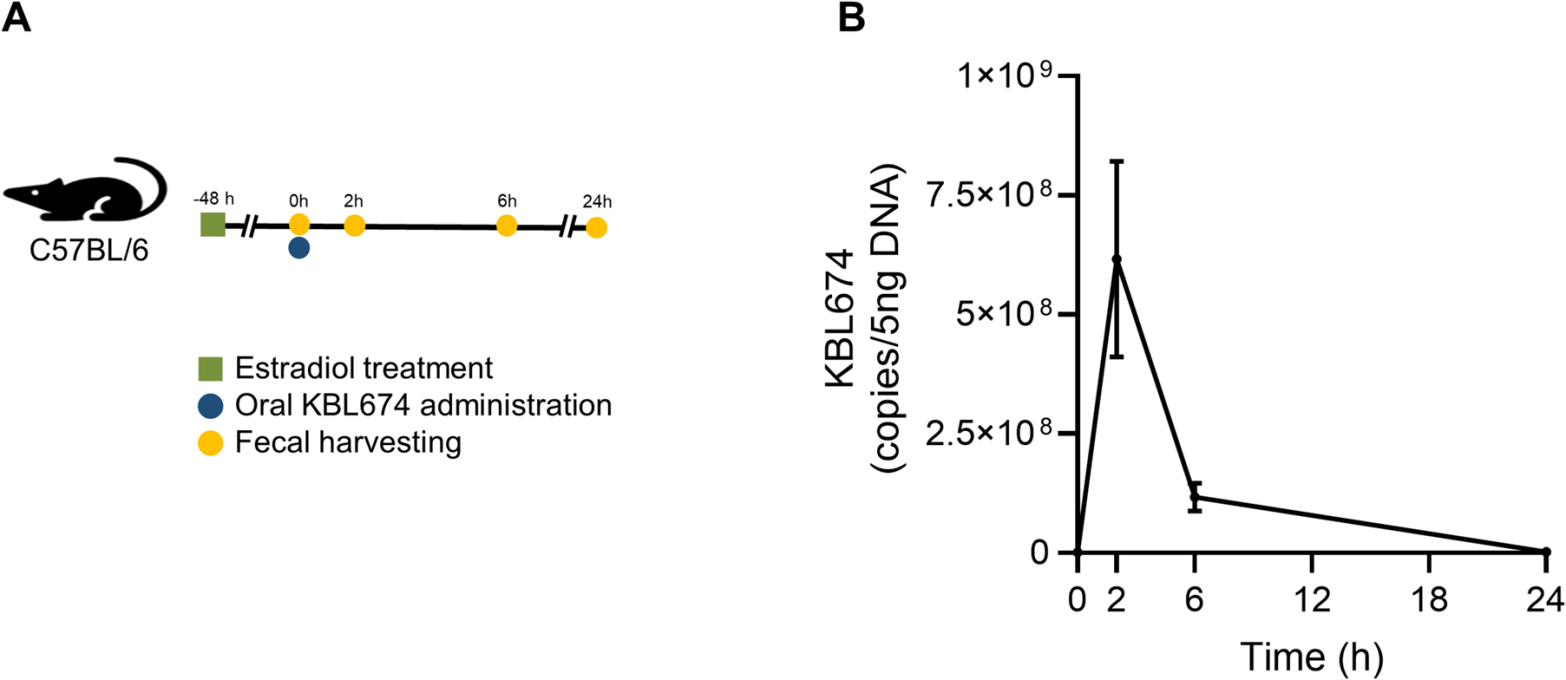
Excretion profiling of *L. fermentum* KBL674 after oral administration. **A** The experimental scheme of the excretion model. **B** Changes of *L. fermentum* KBL674 concentrations in feces. Fecal samples of mice were collected at 0, 2, 6, and 24 h after oral administration of *L. fermentum* KBL674. Concentrations of *L. fermentum* KBL674 were measured by qPCR. Data are expressed as means ± SEM

The level of *L. fermentum* KBL674 DNA in feces peaked at 2 h after oral administration (6.16 × 10^8^ copies per 5 ng DNA) (Fig. 3). *L. fermentum* KBL674 DNA was detectable at 6 h (1.2 × 10^8^ copies per 5 ng DNA) but not at 24 h. No toxicity-related reactions were observed in the mice throughout the entire experiment.

### Effect of *L. fermentum* KBL674 on the Gut Microbiome

**Fig. 4:**
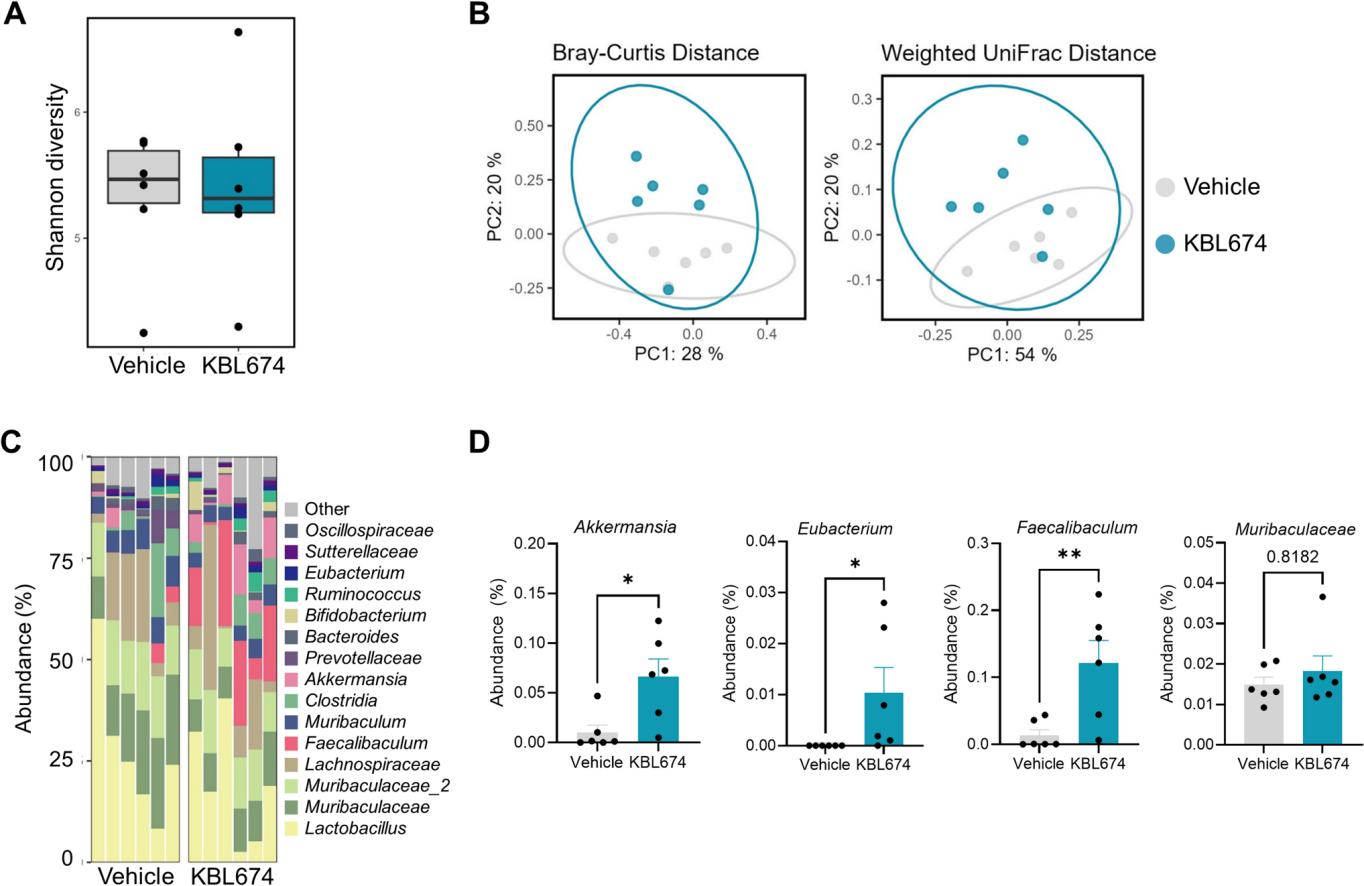
Effect of *L. fermentum* KBL674 on the fecal microbiome of mice with vaginal *C. albicans* infection. **A** Shannon indices for alpha-diversity. **B** PCoA plots with Bray–Curtis or weighted UniFrac distances for beta-diversity. **C** Microbiome composition at the genus level. **D** Abundances in the bacterial genera. When appropriate, data are expressed as means ± SEM. Asterisks indicate statistical significance (*p < 0.05; **p < 0.01; Mann–Whitney *U* test)

**Fig. 5:**
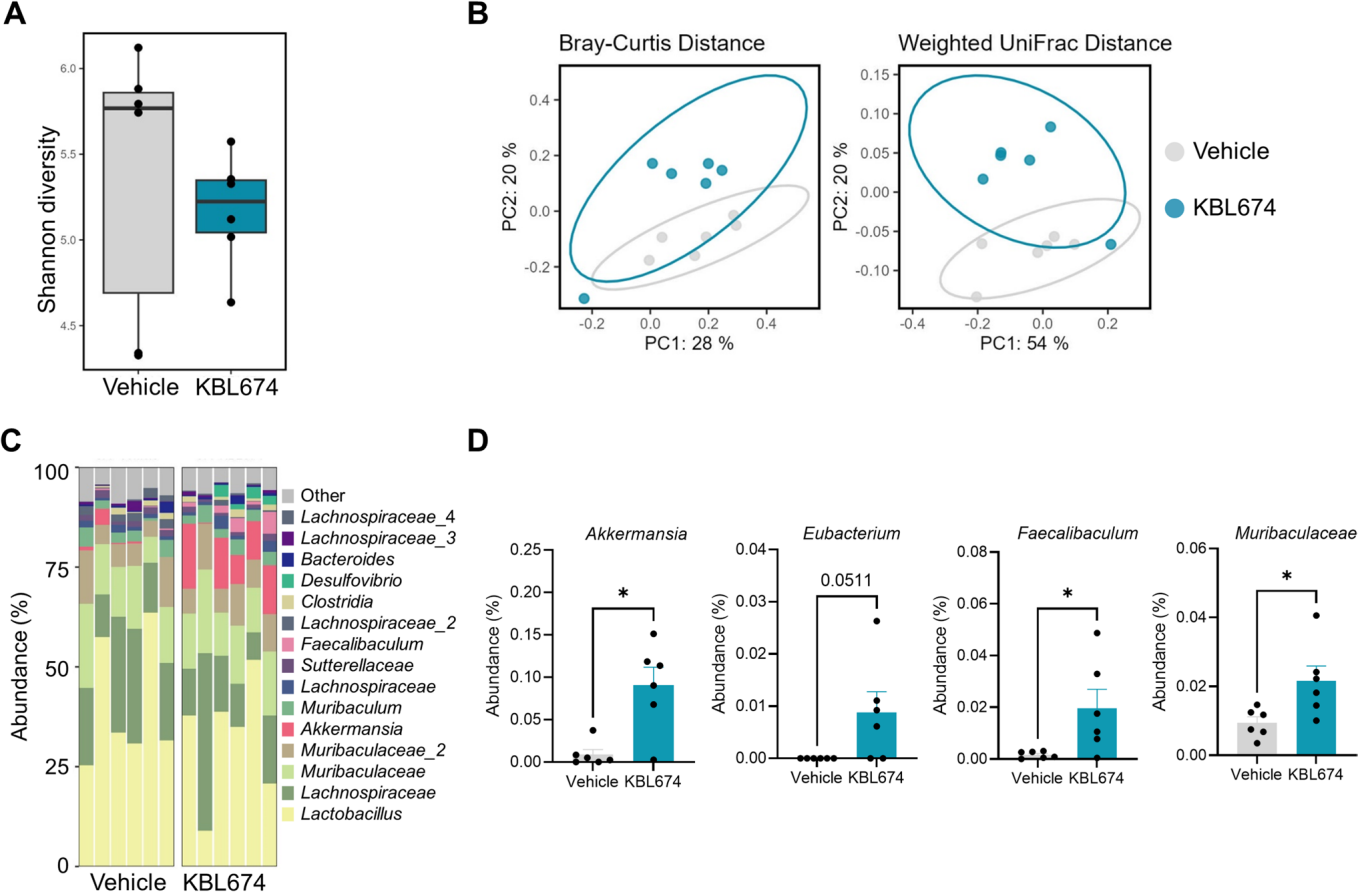
Effect of *L. fermentum* KBL674 on the cecal microbiome of mice with vaginal *C. albicans* infection. **A** Shannon indices for alpha-diversity. **B** PCoA plots with Bray–Curtis or weighted UniFrac distances for beta-diversity. **C** Microbiome composition at the genus level. **D** Abundances in the bacterial genera. When appropriate, data are expressed as means ± SEM. Asterisks indicate statistical significance (*p < 0.05; Mann–Whitney *U* test)

To investigate the effects of *L. fermentum* KBL674 on the gut microbiome, the fecal and cecal microbiome were analyzed (Figs. 4 and 5). Alpha diversities did not significantly differ between the *L. fermentum* KBL674-treated group and the vehicle group (Figs. 4A and 5A). Beta diversities of the fecal and cecal microbiome in the *L. fermentum* KBL674-treated group clustered distinctly compared with the vehicle group (Figs. 4B and 5B). Abundances of the genera *Akkermansia* and *Faecalibaculum* were increased in mice with *L. fermentum* KBL674 (Figs. 4C and 5C). Abundances of the genera *Akkermansia* (*p* < 0.05), *Eubacterium* (*p* < 0.05), and *Faecalibaculum* (*p* < 0.01) were significantly increased in feces from the *L. fermentum* KBL674-treated group compared with the vehicle-treated group (Fig. 4D). Abundances of the genera *Akkermansia* (*p* < 0.05) and *Faecalibaculum* (*p* < 0.05) and family *Muribaculaceae* (*p* < 0.05) were significantly increased in ceca from the *L. fermentum* KBL674-treated group compared with the vehicle-treated group (Fig. 5D). Administration of *L. fermentum* KBL674 did not affect the abundance of the genus *Lactobacillus* within the gut microbiome.

### Correlations between the Vaginal *C. albicans* Burden and Gut Microbiome Composition

**Fig. 6:**
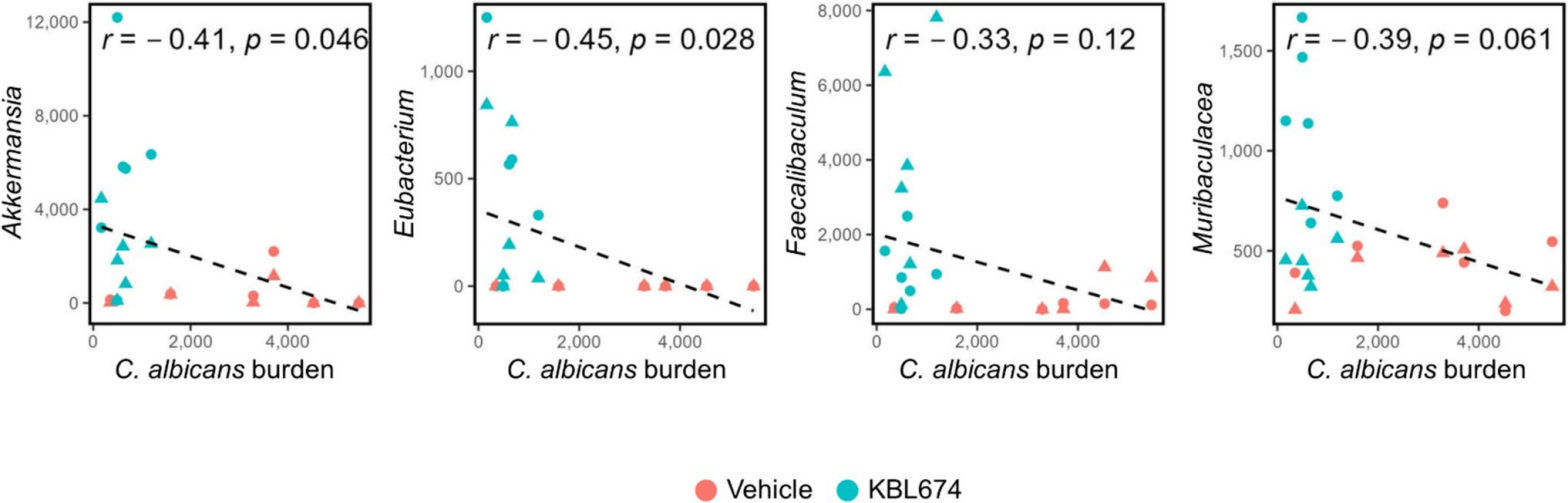
Correlations between the vaginal *C. albicans* burden and gut microbiome composition. Triangles and circles denote fecal and cecal samples, respectively. Pearson’s correlation coefficients (*r*) indicate strengths of correlations. Asterisks indicate statistical significance (*p < 0.05)

Figure 6 shows correlations between the vaginal *C. albicans* burden and gut microbiome composition. The vaginal *C. albicans* burden was negatively correlated with the genera *Akkermansia* (*r* = − 0.41; *p* = 0.046) and *Eubacterium* (*r* = − 0.45; *p* = 0.028). Additionally, the family *Muribaculaceae*, with a significantly increased abundance in the ceca from the *L. fermentum* KBL674-treated group, was negatively correlated with the vaginal *C. albicans* burden (*r* = − 0.38; *p* = 0.063). The abundance of the genus *Faecalibaculum* (r = − 0.33; *p* = 0.12), significantly increased in feces from the *L. fermentum* KBL674-treated group, tended to be negatively correlated with the vaginal *C. albicans* burden.

## Discussion

Inhibitory effects of *Lactobacillus* species, on various pathogenic microorganisms, have been well-reported [25–27]. The composition and function of the vaginal microbiome are important for vaginal health maintenance [28]. A *Lactobacillus-*dominated vaginal microbiome is strongly correlated with vaginal pH and the Nugent score, which is a diagnostic method for bacterial vaginosis [29]. Our previous study also revealed that the supernatant originated from *L. fermentum* KBL674 significantly inhibited the growth and hyphal transition of *C. albicans*, indicating that vaginal *Lactobacillus* species could be used to prevent vaginal candidiasis [14]. *C. albicans* hyphal growth and the vaginal *C. albicans* burden were significantly reduced in the *L. fermentum* KBL674-treated group (Figs. 1 and 2C). Therefore, *L. fermentum* KBL674 has potential as a probiotic for maintaining vaginal health.

Vaginal candidiasis has several symptoms, including *C. albicans* filamentation and vaginal inflammation. Lactobacilli produce various small molecules that alleviate these symptoms [30]. Oral administration of *L. fermentum* KBL674 reduced tissue thickness and immune-cell infiltration in mice with vaginal candidiasis (Fig. 2D). This finding is consistent with previous in vitro results concerning the abilities of vaginal *Lactobacillus* species to modulate the growth and hyphal morphogenesis of *C. albicans* [31–33]. Further research including a quantitative approach of immune cell profiling is needed regarding the mechanisms by which *L. fermentum* KBL674 inhibits *Candida* in the vagina.

Figure 3 B elucidates the fecal excretion profile of oral administered *L. fermentum* KBL674, indicating that most of *L. fermentum* KBL674 shedding occurs within 6 h via feces. After 24 h of administration, *L. fermentum* KBL674 was not detected in feces (Fig. 3B) and any adverse effects were not observed in mice despite of the high dosage of *L. fermentum* KBL674. Therefore, high concentrations of *L. fermentum* KBL674 can be considered safe via the oral route because *L. fermentum* KBL674 does not stay for a long time in the gut. However, the human study should be performed to elucidate the absorption-distribution-metabolism-excretion profile of *L. fermentum* KBL674 for convincing the safety of oral administration of high concentrations of *L. fermentum* KBL674. Moreover, additional studies applying alternative administration methods (e.g., direct delivery in vagina) also need to be planned with tracking potential adverse effects for further applications of *L. fermentum* KBL674 in *C. albicans* infection.

The gut microbiome of the *L. fermentum* KBL674-treated group exhibited beta diversities that differed from those of the vehicle group (Figs. 4B and 5B). *Lactobacillus* strains modulate the gut microbiome composition in mice with atopic dermatitis and inflammatory bowel disease [34–37]. Moreover, *L. fermentum* KBL674 increased the abundances of the genera *Akkermansia*, *Eubacterium*, and *Faecalibaculum* and the family *Muribaculaceae*, which are associated with gut health (Figs. 4D and 5D). The genus *Akkermansia* enhances anti-inflammatory activities by inducing interleukin-10 production and promoting metabolic health [19, 38–42]. The genus *Eubacterium* is a well-known producer of butyrate, the major short-chain fatty acid in the gut [43]. The genus *Faecalibaculum* is key to maintaining intestinal epithelial homeostasis via modulation of retinoic acid-signaling for controlling eosinophils-oriented interferon-γ [44]. The family *Muribaculaceae* is a dominant bacterial species in the mouse gut [45] and is abundant in mice with administration of *Lactobacillus* species [34, 36]. Intriguingly, despite the administration of *L. fermentum* KBL674, the abundance of the genus *Lactobacillus* did not increase significantly, clearly indicating that *L. fermentum* KBL674 did not settle in the gut. Therefore, *L. fermentum* KBL674 can influence improvements of gut microbiota without actual colonization in the gut.

There were significant negative correlations between the vaginal *C. albicans* burden and the abundances of the genera *Akkermansia* and *Eubacterium* and the family *Muribaculaceae* (Fig. 6). The abundances of these microorganisms were significantly increased in feces and ceca of the *L. fermentum* KBL674-treated group, suggesting associations between the gut microbiome and vaginal *C. albicans* concentration.

Although the gut and vagina are physically separate, they could be indirectly linked through mechanisms such as modulation of immune system or production of effective molecules to inhibit *C. albicans*. Further studies with different dose- or time-dependent approaches (e.g., long-term administration) should be planned to investigate the links between host gut and vagina microbiome, known as the gut-vagina axis, and their effects in preventing vaginal candidiasis fully.

## Conclusion

Oral administration of *L. fermentum* KBL674 has excellent anti-inhibitory capabilities against vaginal *C. albicans* infection in mice. Especially, *L. fermentum* KBL674 reduced *C. albicans* burdens and improved vaginal tissues in mice. Most *L. fermentum* KBL674 was excreted from the gastrointestinal tract within 24 h of administration. *L. fermentum* KBL674 increased abundances of the genera *Akkermansia*, *Eubacterium*, and *Faecalibaculum* and the family *Muribaculaceae*, within the gut. These changes were negatively correlated with the vaginal *C. albicans* burden, indicating links between gut microbiome composition and vaginal *C. albicans* concentration. Overall, our findings suggest that *L. fermentum* KBL674 can directly or indirectly reduce the vaginal *C. albicans* burden and modulate the gut microbiome composition preventively. *L. fermentum* KBL674 could be applied as the probiotics for all women including pregnant or menopause, who have vulnerable conditions for vaginal infection, without serious adverse effects. Moreover, further investigation of the synergistic effects of *L. fermentum* KBL674 with other probiotic strains or conventional treatments will enhance its applications as an effective preventive or therapeutic method for vaginal candidiasis.

## Data Availability

All data supporting the findings of this study are available from the corresponding author upon reasonable request.

## References

[1] Sobel JD (2017) Vulvovaginal candidosis. Lancet 369(9577):1961–1971. 10.1016/S0140-6736(07)60917-9 10.1016/S0140-6736(07)60917-9 17560449

[2] Gonçalves B, Ferreira C, Alves CT, Henriques M, Azeredo J, Silva S (2016) Vulvovaginal candidiasis: epidemiology, microbiology and risk factors. Crit Rev Microbiol 42(6):905–927. 10.3109/1040841X.2015.1091805 26690853 10.3109/1040841X.2015.1091805

[3] Ditte MN, Björn P, Anders H (2013) Use of oral fluconazole during pregnancy and the risk of birth defects. N Engl J Med 369(9):830–839. 10.1056/NEJMoa1301066 23984730 10.1056/NEJMoa1301066

[4] Sobel JD, Wiesenfeld HC, Martens M, Danna P, Hooton TM, Rompalo A, Sperling M, Livengood C 3rd, Horowitz B, Thron JV, Edwards L, Panzer H, Chu T-C (2024) Maintenance fluconazole therapy for recurrent vulvovaginal candidiasis. N Engl J Med 351(9):876–883. 10.1056/NEJMoa033114 10.1056/NEJMoa033114 15329425

[5] Klebanoff SJ, Hillier SL, Eschenbach DA, Waltersdorph AM (1991) Control of the microbial flora of the vagina by H_2_O_2_-generating lactobacilli. J Infect Dis 164(1):94–100. 10.1093/infdis/164.1.94 1647428 10.1093/infdis/164.1.94

[6] Boskey ER, Cone RA, Whaley KJ, Moench TR (2001) Origins of vaginal acidity: high D/L lactate ratio is consistent with bacteria being the primary source. Hum Reprod 16(9):1809–1813. 10.1093/humrep/16.9.1809 11527880 10.1093/humrep/16.9.1809

[7] Voravuthikunchai SP, Bilasoi S, Supamala O (2006) Antagonistic activity against pathogenic bacteria by human vaginal lactobacilli. Anaerobe 12(5):221–226. 10.1016/j.anaerobe.2006.06.003 16931064 10.1016/j.anaerobe.2006.06.003

[8] Gosmann C, Anahtar MN, Handley SA, Farcasanu M, Abu-Ali G, Bowman BA, Padavattan N, Desai C, Droit L, Moodley A, Dong M, Chen Y, Ismail N, Ndungu T, Ghebremichael MS, Wesemann DR, Mitchell C, Dong KL, Huttenhower C, Walker BD, Kwon DS (2017) *Lactobacillus*-deficient cervicovaginal bacterial communities are associated with Increased HIV acquisition in young south African women. Immunity 46(1):29–37. 10.1016/j.immuni.2016.12.013 28087240 10.1016/j.immuni.2016.12.013 PMC5270628

[9] McClelland RS, Lingappa JR, Srinivasan S, Kinuthia J, John-Stewart GC, Jaoko W, Richardson BA, Yuhas K, Fiedler TL, Mandaliya KN, Munch MM, Mugo NR, Cohen CR, Baeten JM, Celum C, Overbaugh J, Fredricks DN (2018) Evaluation of the association between the concentrations of key vaginal bacteria and the increased risk of HIV acquisition in African women from five cohorts: a nested case-control study. Lancet Infect Dis 18(5):554–564. 10.1016/S1473-3099(18)30058-6 29396006 10.1016/S1473-3099(18)30058-6 PMC6445552

[10] DiGiulio DB, Callahan BJ, McMurdie PJ, Costello EK, Lyell DJ, Robaczewska A, Sun CL, Goltsman DSA, Wong RJ, Shaw G, Stevenson DK, Homes SP, Relman DA (2015) Temporal and spatial variation of the human microbiota during pregnancy. Proc Natl Acad Sci U S A 112(35):11060–11065. 10.1073/pnas.1502875112 26283357 10.1073/pnas.1502875112 PMC4568272

[11] Anukam KC, Osazuwa E, Osemene GI, Ehigiagbe F, Bruce AW, Reid G (2006) Clinical study comparing probiotic *Lactobacillus* GR-1 and RC-14 with metronidazole vaginal gel to treat symptomatic bacterial vaginosis. Microbes Infect 8(12):2772–2776. 10.1016/j.micinf.2006.08.008 17045832 10.1016/j.micinf.2006.08.008

[12] Martinez RCR, Franceschini SA, Patta MC, Quintana SM, Gomes BC, De Martinis ECP, Reid G (2009) Improved cure of bacterial vaginosis with single dose of tinidazole (2 g), *Lactobacillus **rhamnosus* GR-1, and *Lactobacillus **reuteri* RC-14: a randomized, double-blind, placebo-controlled trial. Can J Microbiol 55(2):133–138. 10.1139/W08-102 19295645 10.1139/w08-102

[13] Cohen CR, Wierzbicki MR, French AL, Morris S, Newmann S, Reno H, Green L, Miller S, Powell J, Parks T, Hemmerling A (2020) Randomized trial of Lactin-V to prevent recurrence of bacterial vaginosis. N Engl J Med 382(20):1906–1915. 10.1056/NEJMoa1915254 32402161 10.1056/NEJMoa1915254 PMC7362958

[14] Jang SJ, Lee K, Kwon B, You HJ, Ko G (2019) Vaginal lactobacilli inhibit growth and hyphae formation of *Candida **albicans*. Sci Rep 9(1):8121. 10.1038/s41598-019-44579-4 31148560 10.1038/s41598-019-44579-4 PMC6544633

[15] Schneider CA, Rasband WS, Eliceiri KW (2012) NIH image to ImageJ: 25 years of image analysis. Nat Methods 9(7):671–675. 10.1038/nmeth.2089 22930834 10.1038/nmeth.2089 PMC5554542

[16] Yano J, Fidel PL (2011) Protocols for vaginal inoculation and sample collection in the experimental mouse model of *Candida* vaginitis. J Vis Exp 58:3382. 10.3791/3382 10.3791/3382 PMC3369659 22215135

[17] Borghi M, De Luca A, Puccetti M, Jaeger M, Mencacci OV, Pariano M, Garlanda C, Moretti S, Bartoli A, Sobel J, van de Veerdonk FL, Dinarello CA, Netea MG, Romani L (2015) Pathogenic NLRP3 inflammasome activity during *Candida* infection is negatively regulated by IL-22 via activation of NLRC4 and IL-1Ra. Cell Host Microbe 18(2):198–209. 10.1016/j.chom.2015.07.004 26269955 10.1016/j.chom.2015.07.004

[18] Klindworth A, Pruesse E, Schweer T, Peplies J, Quast C, Horn M, Glöckner FO (2013) Evaluation of general 16S ribosomal RNA gene PCR primers for classical and next-generation sequencing-based diversity studies. Nucleic Acids Res 41(1):e1. 10.1093/nar/gks808 22933715 10.1093/nar/gks808 PMC3592464

[19] Yoon HS, Cho CH, Yun MS, Jang SJ, You HJ, Kim J, Han D, Cha KH, Moon SH, Lee K, Kim Y-J, Lee S-J, Nam T-W, Ko G (2021) *Akkermansia **muciniphila* secretes a glucagon-like peptide-1-inducing protein that improves glucose homeostasis and ameliorates metabolic disease in mice. Nat Microbiol 6(5):563–573. 10.1038/s41564-021-00880-5 33820962 10.1038/s41564-021-00880-5

[20] Singer E, Bushnell B, Coleman-Derr D, Bowman B, Bowers RM, Levy A, Gies EA, Cheng JF, Copeland A, Klenk HP, Hallam SJ, Hugenholtz P, Tringe SG, Woyke T (2016) High-resolution phylogenetic microbial community profiling. ISME J 10(8):2020–2032. 10.1038/ismej.2015.249 26859772 10.1038/ismej.2015.249 PMC5029162

[21] Bolger AM, Lohse M, Usadel B (2014) Trimmomatic: a flexible trimmer for Illumina sequence data. Bioinformatics 30(15):2114–2120. 10.1093/bioinformatics/btu170 24695404 10.1093/bioinformatics/btu170 PMC4103590

[22] Bolyen E, Rideout JR, Dillon MR, Bokulich NA, Abnet CC, Al-Ghalith GA, Alexander H, Alm EJ, Arumugam M, Asnicar F, Bai Y, Bisanz JE, Bittinger K, Brejnrod A, Brislawn CJ, Brown CT, Callahan BJ, Caraballo-Rodríguez AM, Chase J, Cope EK, Da Silva R, Diener C, Dorrestein PC, Douglas GM, Durall DM, Duvallet C, Edwardson CF, Ernst M, Estaki M, Fouquier J, Gauglitz JM, Gibbons SM, Gibson DL, Gonzalez A, Gorlick K, Guo J, Hillmann B, Holmes S, Holste H, Huttenhower C, Huttley GA, Janssen S, Jarmusch AK, Jiang L, Kaehler BD, Kang KB, Keefe CR, Keim P, Kelley ST, Knights D, Koester I, Kosciolek T, Kreps J, Langille MGI, Lee J, Ley R, Liu YX, Loftfield E, Lozupone C, Maher M, Marotz C, Martin BD, McDonald D, McIver LJ, Melnik AV, Metcalf JL, Morgan SC, Morton JT, Naimey AT, Navas-Molina JA, Nothias LF, Orchanian SB, Pearson T, Peoples SL, Petras D, Preuss ML, Pruesse E, Rasmussen LB, Rivers A, Robeson MS 2nd, Rosenthal P, Segata N, Shaffer M, Shiffer A, Sinha R, Song SJ, Spear JR, Swafford AD, Thompson LR, Torres PJ, Trinh P, Tripathi A, Turnbaugh PJ, Ul-Hasan S, van der Hooft JJJ, Vargas F, Vázquez-Baeza Y, Vogtmann E, von Hippel M, Walters W, Wan Y, Wang M, Warren J, Weber KC, Williamson CHD, Willis AD, Xu ZZ, Zaneveld JR, Zhang Y, Zhu Q, Knight R, Caporaso JG (2019) Reproducible, interactive, scalable and extensible microbiome data science using QIIME 2. Nat Biotechnol 37(9):852–857. 10.1038/s41587-019-0209-9 31341288 10.1038/s41587-019-0209-9 PMC7015180

[23] Quast C, Pruesse E, Yilmaz P, Gerken J, Schweer T, Yarza P, Peplies J, Glöckner FO (2013) The SILVA ribosomal RNA gene database project: improved data processing and web-based tools. Nucleic Acids Res 41(D1):D590–D596. 10.1093/nar/gks1219 23193283 10.1093/nar/gks1219 PMC3531112

[24] Bisanz JE (2020) qiime2R: importing QIIME2 artifacts and associated data into R sessions. GitHub. https://github.com/jbisanz/qiime2R. Accessed 17 Jul 2024

[25] Vasiee A, Behbahani BA, Yazdi FT, Mortazavi SA, Noorbakhsh H (2018) Diversity and probiotic potential of lactic acid bacteria isolated from Horreh, a traditional Iranian fermented food. Probiotics Antimicro Prot 10:258–268. 10.1007/s12602-017-9282-x 10.1007/s12602-017-9282-x 28527125

[26] Falah F, Vasiee A, Behbahani BA, Yazdi FT, Moradi S, Mortazavi SA, Roshanak S (2019) Evaluation of adherence and anti-infective properties of probiotic *Lactobacillus fermentum* strain 4–17 against *Escherichia coli* causing urinary tract infection in humans. Microb Pathog 131:246–253. 10.1016/j.micpath.2019.04.006 30974159 10.1016/j.micpath.2019.04.006

[27] Rahmati-Joneidabad M, Behbahani BA, Taki M, Hesarinejad MA, Toker OS (2024) Evaluation of the probiotic, anti-microbial, anti-biofilm, and safety properties of *Levilactobacillus brevis* Lb13H. LWT 207:116636. 10.1016/j.lwt.2024.116636

[28] Anahtar MN, Gootenberg DB, Mitchell CM, Kwon DS (2018) Cervicovaginal microbiota and reproductive health: the virtue of simplicity. Cell Host Microbe 23(2):159–168. 10.1016/j.chom.2018.01.013 29447695 10.1016/j.chom.2018.01.013

[29] Ravel J, Gajer P, Abdo Z, Schneider GM, Koenig SSK, McCulle SL, Karlebach S, Gorle R, Jussell J, Tacket CO, Brotman RM, Davis CC, Ault K, Peralta L, Forney L (2011) Vaginal microbiome of reproductive-age women. Proc Natl Acad Sci U S A 108(supplement_1):4680–4687. 10.1073/pnas.1002611107 10.1073/pnas.1002611107 PMC3063603 20534435

[30] Glick VJ, Webber CA, Simmons LE, Martin MC, Ahmad M, Kim CH, Adams AND, Bang S, Chao MC, Howard NC, Fortune SM, Verma M, Jost M, Beura LK, James MJ, Lee SY, Mitchell CM, Clardy J, Kim KH, Gopinath S (2024) Vaginal lactobacilli produce anti-inflammatory β-carboline compounds. Cell Host Microbe 32(11):1897–1909.e7. 10.1016/j.chom.2024.09.014 10.1016/j.chom.2024.09.014 PMC11694765 39423813

[31] Wang S, Wang Q, Yang E, Yan L, Li T, Zhuang H (2017) Antimicrobial compounds produced by vaginal *Lactobacillus **crispatus* are able to strongly inhibit *Candida **albicans* growth, hyphal formation and regulate virulence-related gene expressions. Front Microbiol 8:564. 10.3389/fmicb.2017.00564 28421058 10.3389/fmicb.2017.00564 PMC5378977

[32] Emily M, Christopher D, Leighann S, Kean R, Williams S, Metcalfe R, Thomas R, Richardson R, Gerasimidis K, Nile CJ, Williams C, Ramage G (2021) Recurrent vulvovaginal candidiasis: a dynamic interkingdom biofilm disease of *Candida* and *Lactobacillus.* mSystems 6(4): e0062221. 10.1128/msystems.00622-21 10.1128/mSystems.00622-21 PMC8407231 34374560

[33] Alonso-Roman R, Last A, Mirhakkak MH, Sprague JL, Möller L, Großmann P, Graf K, Gratz R, Mogavero S, Vylkova S, Panagiotou G, Schäuble S, Hube B, Gresnigt MS (2022) *Lactobacillus **rhamnosus* colonisation antagonizes *Candida **albicans* by forcing metabolic adaptations that compromise pathogenicity. Nat Commun 13(1):3192. 10.1038/s41467-022-30661-5 35680868 10.1038/s41467-022-30661-5 PMC9184479

[34] Jang YJ, Kim W-K, Han DH, Lee K, Ko G (2019) *Lactobacillus **fermentum* species ameliorate dextran sulfate sodium-induced colitis by regulating the immune response and altering gut microbiota. Gut Microbes 10(6):696–711. 10.1080/19490976.2019.1589281 30939976 10.1080/19490976.2019.1589281 PMC6866707

[35] Kim W-K, Jang YJ, Han DH, Jeon K, Lee C, Han HS, Ko G (2020) *Lactobacillus **paracasei* KBL382 administration attenuates atopic dermatitis by modulating immune response and gut microbiota. Gut Microbes 12(1):1819156. 10.1080/19490976.2020.1819156 33016202 10.1080/19490976.2020.1819156 PMC7553742

[36] Kim W-K, Han DH, Jang YJ, Park SJ, Jang SJ, Lee G, Han HS, Ko G (2021) Alleviation of DSS-induced colitis via *Lactobacillus acidophilus* treatment in mice. Food Funct 12(1):340–350. 10.1039/D0FO01724H 33325946 10.1039/d0fo01724h

[37] Kim W-K, Jang YJ, Park S, Min S, Kwon H, Jo MJ, Ko G (2024) *Lactobacillus acidophilus* KBL409 ameliorates atopic dermatitis in a mouse model. J Microbiol 62(2):91–99. 10.1007/s12275-024-00104-5 38386273 10.1007/s12275-024-00104-5 PMC11021314

[38] Schneeberger M, Everard A, Gómez-Valadés AG, Matamoros S, Ramírez S, Delzenne NM, Gomis R, Claret M, Cani PD (2015) *Akkermansia **muciniphila* inversely correlates with the onset of inflammation, altered adipose tissue metabolism and metabolic disorders during obesity in mice. Sci Rep 5:16643. 10.1038/srep16643 26563823 10.1038/srep16643 PMC4643218

[39] Li J, Lin S, Vanhoutte PM, Woo CW, Xu A (2016) *Akkermansia**muciniphila* protects against atherosclerosis by preventing metabolic endotoxemia-induced inflammation in Apoe^−/−^ Mice. Circulation 133(24):2434–2446. 10.1161/CIRCULATIONAHA.115.019645 27143680 10.1161/CIRCULATIONAHA.115.019645

[40] Ottman N, Reunanen J, Meijerink M, Pietilä TE, Kainulainen V, Klievink J, Huuskonen L, Aalvink S, Skurnik M, Boeren S, Satokari R, Mercenier A, Palva A, Smidt H, de Vos WM, Belzer C (2017) Pili-like proteins of *Akkermansia **muciniphila* modulate host immune responses and gut barrier function. PLoS ONE 12(3):e0173004. 10.1371/journal.pone.0173004 28249045 10.1371/journal.pone.0173004 PMC5332112

[41] Grajeda-Iglesias C, Durand S, Daillère R, Iribarren K, Lemaitre F, Derosa L, Aprahamian F, Bossut N, Nirmalathasan N, Madeo F, Zitvogel L, Kroemer G (2021) Oral administration of *Akkermansia muciniphila* elevates systemic antiaging and anticancer metabolites. Aging 13(5):6375–6405. 10.18632/aging.202739 10.18632/aging.202739 PMC7993698 33653967

[42] Si J, Kang H, You HJ, Ko G (2022) Revisiting the role of * Akkermansia ** muciniphila * as a therapeutic bacterium. Gut Microbes 14(1):2078619. 10.1080/19490976.2022.2078619 35613313 10.1080/19490976.2022.2078619 PMC9135416

[43] Ghosh S, Pramanik S (2021) Structural diversity, functional aspects and future therapeutic applications of human gut microbiome. Arch Microbiol 203(9):5281–5308. 10.1007/s00203-021-02516-y 34405262 10.1007/s00203-021-02516-y PMC8370661

[44] Cao YG, Bae S, Villarreal J, Moy M, Chun E, Michaud M, Lang JK, Glickman JN, Lobel L, Garrett WS (2022) *Faecalibaculum rodentium* remodels retinoic acid signaling to govern eosinophil-dependent intestinal epithelial homeostasis. Cell Host Microbe 30(9):1295–1310. 10.1016/j.chom.2022.07.015 35985335 10.1016/j.chom.2022.07.015 PMC9481734

[45] Lagkouvardos I, Lesker TR, Hitch TCA, Gálvez EJC, Smit N, Neuhaus K, Wang J, Baines JF, Abt B, Stecher B, Overmann J, Strowig T, Clavel T (2019) Sequence and cultivation study of *Muribaculaceae* reveals novel species, host preference, and functional potential of this yet undescribed family. Microbiome 7(1):28. 10.1186/s40168-019-0637-2 30782206 10.1186/s40168-019-0637-2 PMC6381624

